# Effects of dietary energy levels on rumen bacterial community composition in Holstein heifers under the same forage to concentrate ratio condition

**DOI:** 10.1186/s12866-018-1213-9

**Published:** 2018-07-11

**Authors:** Yanliang Bi, Shuqin Zeng, Rong Zhang, Qiyu Diao, Yan Tu

**Affiliations:** Key Laboratory of Feed Biotechnology of the Ministry of Agriculture, Feed Research Institute, Chinese Academy of Agricultural Sciences, NO. 12 Zhongguancun South Street, Haidian District, Beijing, 100081 China

**Keywords:** Dietary energy level, Forage to concentrate ratio, Heifer, Rumen bacterial community, Sequencing

## Abstract

**Background:**

The rumen bacterial community plays a critical role in feeds degradation and productivity. The effects of different forage to concentrate ratios on the ruminal microbial population structure have been studied extensively; however, research into changes in the ruminal bacterial community composition in heifers fed different energy level diets, with the same forage to concentrate ratio, has been very limited. The purpose of this study was to investigate the effects of different dietary energy levels, with the same forage to concentrate ratio, on ruminal bacterial community composition of heifers. Furthermore, we also determine the relationship between rumen bacteria and ruminal fermentation parameters.

**Results:**

The 16S rRNA gene sequencing showed that, under the same forage to concentrate ratio of 50:50, an 8% difference in dietary energy level had no significant impact on the alpha diversity and the relative abundance of the major phyla and most of the major genera in heifers. In all the treatments groups, *Firmicutes*, *Bacteroidetes*, and *Proteobacteria* were the dominant phyla. Spearman correlation analysis between the relative abundances of the rumen bacteria at the genus level and the fermentation parameters showed that the relative abundances of *Prevotella* and BF311 were positively correlated with the ammonia nitrogen and butyrate concentrations, and these two genera were negatively correlated with the propionate and isovalerate concentrations, respectively, and the genus *Bifidobacterium* was positively correlated with the butyrate concentration and was negatively correlated with propionate and isovalerate concentration. The total volatile fatty acid concentration was positively correlated with BF311 abundances, and was negatively correlated with *Trichococcus* and *Facklamia* abundances.

**Conclusions:**

Under the same forage to concentrate ratio condition of 50:50, an 8% difference in dietary energy levels had little impact on rumen bacterial community composition in heifers. The correlations between some genera of ruminal bacteria and the concentrations of volatile fatty acids and ammonia nitrogen might be indicative that the ruminal fermentation parameters are strongly influenced by the rumen bacterial community composition.

## Background

The rumen is an extremely complex microbial ecosystem, and contains a great diversity of bacteria, archaea, viruses, protozoa, and fungi [1]. The ruminal microorganisms play an important role in degrading complex feeds into volatile fatty acids (VFA) and ammonia, and synthesizing vitamin B and microbial cell protein, which are critical for animal health and production performance [2–4].

The structure of the ruminal microbial community is influenced by several factors, such as age, diet, health status, host species, geographical location, and whether the host has received antibiotic treatment [5, 6]. Diet is a major factor that determines rumen community structure and microbial fermentation patterns [7–9]. For example, during adaptation to a high-grain diet from a high-forage diet, significant changes in the rumen bacterial population structure and major fermentation products have been reported [10–12]. A forage-based diet is dominated by cellulolytic and fibrolytic bacteria, which degrade the cellulose and hemicellulose, while a concentrate-based diet is dominated by starch-degrading amylolytic bacteria, which ferment the starch and sugars. Diet has a clear effect in shaping the ruminal microbial community; meanwhile, the structure of the ruminal bacterial community has been proven to have a correlation with feed efficiency [8, 13]. A previous study showed that changes in the ruminal microbial population structure could help promote feed efficiency and mean daily gain in cattle [14].

In the feedlot cattle industry, it is common to improve mean daily gain and production performance by increasing the dietary energy density. Two prevalent methods widely used to increase the dietary energy density are changing the forage to concentrate ratio and changing the dietary energy levels under the same forage to concentrate ratio condition. A change in the forage to concentrate ratio is direct and allows for increasing the dietary energy density to a great extent. However, when a forage diet is abruptly changed to a high-grain diet, a rapid decrease in ruminal pH due to lactic acid production has been reported [15, 16], which may lead to digestive disorders such as ruminal acidosis [14, 17, 18]. Ruminal acidosis is an important example of an interaction between ruminal microorganism and diet that can impair animal health and production [19, 20]. The change in dietary energy levels under the same forage to concentrate ratio condition is a limited increase in the diet energy density, thus giving few opportunities to cause ruminal acidosis.

Understanding the dynamic ruminal microbial community and its functions is essential to facilitate feed management practices that improve optimal production efficiency [9, 11]. Many studies have examined the effects of different forage to concentrate ratios on the rumen microbial population structure [7, 10–12]. However, there is little published information on the effects of different dietary energy levels with the same forage to concentrate ratio on the ruminal bacterial community composition in cattle. Therefore, the aim of this study was to investigate the sequential dynamic changes in bacterial community composition of heifers fed different energy level diets with the same forage to concentrate ratio using next-generation sequencing technologies, and to explore their relationships with ruminal fermentation parameters. The hypothesis was that under the same forage to concentrate ratio condition, different dietary energy levels would significantly affect the rumen bacterial community composition of Holstein heifers.

## Methods

### Animal experiments and sample collection

The experimental procedures used in this study were approved by the Animal Ethics Committee of the Chinese Academy of Agricultural Sciences and were performed in accordance with good scientific practices and national legislation.

Twelve Chinese Holstein heifers (7 months old; body weight 268.10 ± 7.32 kg) fed the same diet were selected from a commercial dairy farm and randomly divided into three dietary treatments with four heifers each. The heifers were fed total mixed rations (Table 1) with 9.34 (low, L), 10.08 (medium, M), and 10.88 (high, H) MJ/kg dry matter of metabolizable energy for 90 days in a same season. The three kinds of diets had the same forage to concentrate ratio of 50:50. The dietary energy level of the M group was formulated according to the Nutrient Requirements of Dairy Cattle [21] recommendations, and the dietary energy levels of the L and H groups were 92 and 108% of the M group, respectively. In this study, with the same ingredients and the same forage to concentrate ratio, but without fat supplementation, 8% difference of dietary energy level was the upper limit we could formulate between the two adjacent diets, so we choose these three different energy levels. The amount of feed for the three treatments was the same and was calculated to be 2.6% of the mean weight of the M group. The feed intake of each heifer was recorded daily. Clean fresh water was provided ad libitum throughout the study.

**Table 1:** Composition and nutrient levels of the experimental diets (on a dry matter basis)

Items	Treatments		
	L	M	H
Ingredient			
Chinese wildrye	12.50	12.50	12.50
Dry alfalfa hay	16.50	16.50	16.50
Corn silage	21.00	21.00	21.00
Corn	12.90	18.12	27.85
Wheat bran	12	10	8.29
Soybean meal	5	4.44	4
DDGS ^a^	5	5	4.86
Cottonseed meal	4.02	3	3
Rice hull powder	9.08	7.44	0
Premix^b^	2.00	2.00	2.00
Total	100.00	100.00	100.00
Nutrient level			
Dry matter (%)	89.43	89.45	89.64
Metabolizable energy^c^(MJ/kg)	9.34	10.08	10.88
CP^d^ (%)	14.09	14.06	14.12
NDF ^d^ (%)	50.52	48.27	42.74
ADF ^d^ (%)	35.13	33.41	29.19
Ash (%)	7.61	6.93	5.69
Calcium (%)	0.97	0.94	0.92
Phosphorous (%)	0.46	0.46	0.46

^a^DDGS, distillers dried grains with soluble

^b^Manufactured by the Precision Animal Nutrition Research Centre, Beijing, China. Premix provided the following per kg of concentrate: vitamin A, 30150 IU; vitamin D, 9675 IU; vitamin E, 76.5 mg; Fe, 132 mg; Cu, 26 mg; Mn, 118.30 mg; Zn, 166 mg; Se, 0.07 mg; I, 0.11 mg; Co, 0.03 mg; Ca, 6.76 g; P, 0.68 g

^c^Metabolizable energy was calculated according to the Nutrient Requirements of Dairy Cattle (NRC, 2001)

^d^*CP*, Crude protein; *NDF*, Neutral detergent fiber; *ADF*, Acid detergent fiber

At 240, 270, and 300 days of age, 2 h after the morning feeding, a rumen sample (both solid and liquid fractions) was extracted using esophageal tubing as described by Paz et al. [22]. Immediately after collection, 2 ml of the rumen sample was taken and stored in liquid nitrogen until needed for DNA extraction, and the rest of the rumen sample was filtered through four layers of sterile cheesecloth. A filtered liquid sample of 10 ml was collected, acidified with 2 ml 25% (*w*/*v*) metaphosphoric acid, and stored at − 20 °C for analysis of ruminal fermentation parameters.

### Deoxyribonucleic acid (DNA) extraction, amplification, and sequencing

Bacterial DNA was extracted from the rumen samples using an E.Z.N.A.® Stool DNA kit (Omega Bio-tek, Norcross, GA, USA). The quality and quantity of the DNA were measured using an ND 1000 spectrophotometer (NanoDrop Technologies Inc., Wilmington, DE, USA). Conventional polymerase chain reaction (PCR) was performed to amplify the V3–V4 regions of the 16S ribosomal ribonucleic acid (rRNA) gene using universal primers 338F 5′-barcode-ACTCCTACGGGAGGCAGCAG-3′ and 806R 5′- barcode-GGACTACHVGGGTWTCTAAT-3′ [23], where the barcode is an eight-base sequence unique to each sample. PCR was performed in triplicate 50 μl reactions containing 30 ng DNA template, 2 μl of each primer (10 μM), 4 μl 2.5 mM deoxyribonucleotides triphosphate (dNTPs), 5 μl 10 × *Pyrobest* Buffer, 0.3 μl *Pyrobest* DNA Polymerase (2.5 U/μl; TaKaRa Bio Inc., Kusatsu, Shiga, Japan; code DR005A), and 36.7 μl double distilled H_2_O (ddH_2_O). Reaction conditions consisted of: an initial cycle of 95 °C for 5 min; followed by 25 cycles of 95 °C for 30 s, 56 °C for 30 s, and 72 °C for 40 s; and a final extension of 72 °C for 10 min. PCR products were excised from 2% agarose gels, purified using an AxyPrep DNA Gel Extraction kit (Axygen Biosciences, Union City, CA, USA) and quantified using the QuantiFluor™-ST system (Promega, Madison, WI, USA). Equimolar amounts of the barcoded V3–V4 amplicons were pooled and paired-end sequenced on an Illumina MiSeq PE300 platform (Illumina, Inc., San Diego, CA, USA).

### Sequencing data processing and analysis

Raw data of sequencing were filtered through a quality control pipeline using Trimmomatic [24]. In brief, the 300 base pair (bp) reads were truncated at any site receiving a mean quality score of < 20 over a 50 bp sliding window, and any read containing barcode/primer errors or ambiguous character was discarded. Only sequences that overlapped > 10 bp and had < 10% mismatches were assembled. Barcodes and adaptor sequences were then removed from the assembled sequences. Chimeric sequences were detected using USEARCH and removed [25]. The assembled sequences were trimmed of primers and were assigned to operational taxonomic units (OTUs) at a 97% identity threshold using UPARSE [26]. Taxonomy classifications were assigned against the SILVA bacteria alignment database [27] using the Ribosomal Database Project (RDP) classifier [28] with a 0.8 confidence threshold. Sequences were aligned by Python Nearest Alignment Space Termination (PyNAST) [29], and a phylogenetic tree was constructed using FastTree [30].

Alpha diversity values for rumen bacterial communities of different treatments were calculated by various diversity indices (the number of OTUs, the ACE and Chao1 estimator, and the Shannon index) and by normalizing the number of reads in all samples to 13,010 sequences using mothur [31].

### Ruminal fermentation parameters analysis

Ruminal VFA were measured using a gas chromatograph (GC522: Wufeng Instruments, Shanghai, China) that was equipped with a hydrogen flame ionization detector and a 15 ml semi-capillary glass column (0.53 mm in diameter). Ruminal ammonia nitrogen (NH_3_-N) was measured as described previously [32].

### Statistical analyses

The relative abundances of communities at the phylum and genus levels, the alpha diversity indices, and the ruminal fermentation parameters were assessed by analysis of variance using the MIXED procedure of SAS (version 9.2; SAS Institute Inc., Cary, NC, USA). The model included the fixed effects of treatment and age, interaction between treatment and age, and the random effect of the individual nested within treatment. Statistical differences among the means of the treatments were compared using Duncan’s Multiple Range Test. Treatment differences with *P* < 0.05 were considered statistically significant, and 0.05 ≤ *P* < 0.10 was designated as a tendency.

Spearman’s rank correlations between the relative abundances of rumen bacterial community components and fermentation parameters were analyzed using the PROC CORR procedure of SAS. Only genera with a relative abundance ≥0.1% in all samples were included in the analysis. *P* < 0.05 was considered to be a significant correlation.

## Results

### Sequencing depth, coverage, and alpha diversity

**Fig. 1:**
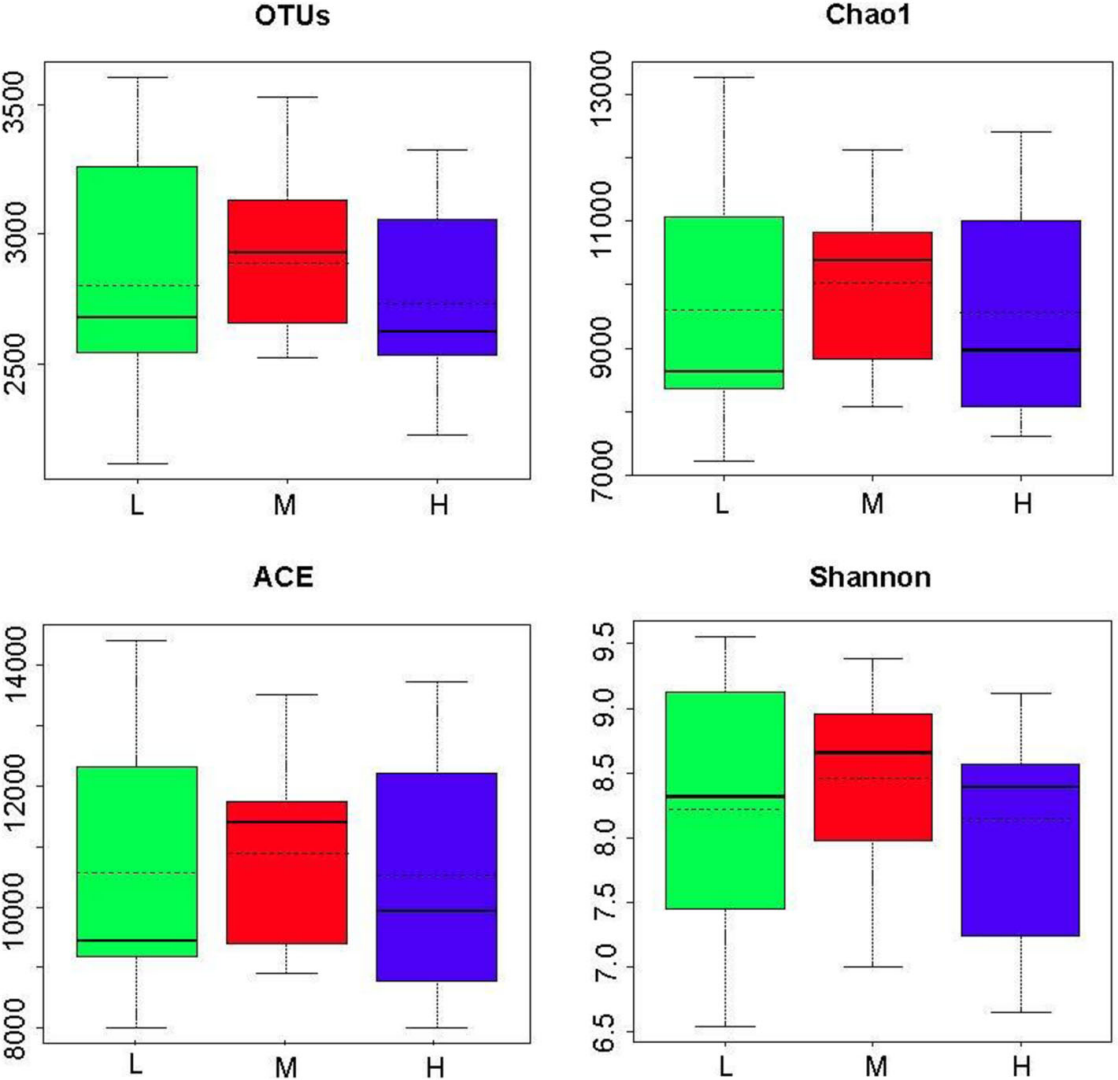
Box plot showing the alpha diversity of the rumen bacterial communities in heifers given different dietary energy levels. Boxes represent the interquartile range between the first and third quartiles and the horizontal full lines inside the boxes define the median. The broken lines inside the boxes represent the mean value. Whiskers represent the maximum and minimum values within 1.5 times the interquartile range from the first and third quartiles, respectively

After data filtering, quality control, and chimera removal, a total of 3,466,038 V3–V4 16S rRNA sequence reads from the 36 samples were generated, with a mean of 96,279 sequence reads for each sample (minimum, 13,013; maximum, 276,932). The mean length of the sequence reads was 435 bp. The overall number of OTUs detected by the analysis was 16,927 based on ≥97% nucleotide sequence identity between reads. With a subsample of 13,010 reads for every sample, the sample-based rarefaction curves showed that our sequencing depth provided sufficient diversity coverage to accurately describe the bacterial composition of all groups. Alpha diversity measures were indicative that, under the same forage to concentrate ratio condition, an 8% difference in dietary energy level had little effect on the number of OTUs, the ACE and Chao1 estimator, and the Shannon index (Figure 1).

### Rumen bacterial community composition across different dietary treatments

At the phylum level, 42 phyla were identified in the rumen samples. Among these 42 phyla, *Firmicutes*, *Bacteroidetes*, and *Proteobacteria* were detected as the dominant phyla regardless of group (Table 2). In addition to these three phyla, the phyla TM7, *Tenericutes*, *Actinobacteria*, *Chloroflexi*, and *Spirochaetes* had a relative abundance of ≥0.1% in all groups. However, the relative abundance and composition of these predominant phyla varied among the groups. At the genus level, 346 classifiable genera were detected in all samples. In total, 26 of these genera showed a relative abundance of ≥0.1% in all samples across the different groups, but their relative levels of abundance were different (Table 3).

**Table 2:** Phylum-level composition of the rumen samples from different groups of heifers (relative abundance ≥0.1%)

Phylum	Treatments			SEM	*P*		
	L	M	H		Treatment	Age	Treatment × Age
*Actinobacteria*	1.97	1.63	1.33	0.0010	0.0690	0.0014	0.0989
*Bacteroidetes*	20.8	23.44	18.69	0.0114	0.2825	< 0.0001	0.9667
*Chloroflexi*	0.29	0.24	0.21	0.0003	0.1783	0.0004	0.1384
*Firmicutes*	54.69	54.79	51.62	0.0065	0.1295	< 0.0001	0.3535
*Proteobacteria*	13.41	10.76	15.57	0.0205	0.6438	0.0002	0.6974
*Spirochaetes*	0.10	0.10	0.08	0.0001	0.7489	0.0001	0.3636
*Tenericutes*	2.03	1.99	2.04	0.0017	0.9897	< 0.0001	0.1050
TM7	2.42	2.1	2.04	0.0016	0.6041	0.0191	0.3990

**Table 3:** Genus-level composition of the rumen samples from different groups of heifers (relative abundance ≥0.1%)

Phylum	Genus	Treatments			SEM	*P*		
		L	M	H		Treatment	Age	Treatment × Age
*Actinobacteria*	*Bifidobacterium*	0.55	0.35	0.17	0.0008	0.1776	0.0202	0.0603
*Bacteroidetes*	CF231	0.65	0.74	0.64	0.0004	0.5347	0.0003	0.2984
	*Prevotella*	4.63	5.96	4.41	0.0029	0.1140	< 0.0001	0.9769
*Chloroflexi*	SHD-231	0.29^a^	0.23^b^	0.21^b^	0.0001	0.0249	0.0007	0.4629
*Firmicutes*	*Anaerostipes*	0.17	0.21	0.18	0.0002	0.7527	0.5715	0.7874
	*Anaerovibrio*	0.24	0.31	0.29	0.0002	0.4666	0.0845	0.6887
	*Blautia*	0.17	0.33	0.29	0.0003	0.0994	< 0.0001	0.0309
	*Butyrivibrio*	2.73	2.67	2.52	0.0010	0.6909	< 0.0001	0.0085
	*Carnobacterium*	9.62	9.08	7.48	0.0193	0.8961	0.0258	0.9839
	*Clostridium*	0.25	0.26	0.19	0.0002	0.2906	0.0230	0.5487
	*Coprococcus*	0.32	0.23	0.17	0.0002	0.1160	< 0.0001	0.5423
	*Facklamia*	0.19	0.12	0.06	0.0003	0.2606	0.0333	0.1067
	L7A_E11	0.12	0.13	0.13	0.0001	0.7989	0.9725	0.4593
	*Mogibacterium*	0.84	0.78	0.86	0.0005	0.8112	0.0004	0.8287
	*Oscillospira*	0.39	0.39	0.35	0.0002	0.6750	0.0046	0.5703
	p-75-a5	0.27	0.25	0.29	0.0002	0.6309	0.0010	0.5379
	RFN20	0.11	0.10	0.11	0.0001	0.9604	< 0.0001	0.7839
	*Ruminococcus*	4.36	3.73	4.04	0.0023	0.5613	< 0.0001	0.6957
	*Selenomonas*	0.09	0.13	0.11	0.0001	0.1286	0.2349	0.4714
	*Shuttleworthia*	0.15	0.16	0.15	0.0001	0.8867	< 0.0001	0.4508
	*Succiniclasticum*	3.48^b^	3.89^ab^	4.49^a^	0.0009	0.0034	0.0090	0.6174
	*Trichococcus*	0.20	0.13	0.12	0.0003	0.6035	0.0018	0.2963
*Proteobacteria*	*Acinetobacter*	0.94	0.49	0.32	0.0017	0.3587	0.0032	0.3414
	*Desulfovibrio*	0.17^ab^	0.20^a^	0.13^b^	0.0001	0.0468	< 0.0001	0.1780
	*Pseudomonas*	0.22	0.12	0.13	0.0003	0.4600	0.2161	0.3478
	*Psychrobacter*	0.27^b^	0.27^b^	0.43^a^	0.0001	< 0.0001	0.0131	< 0.0001

^a,b^Values in the same row with different superscript letters differ significantly (*P* < 0.05)

As shown in Tables 2 and 3, under the same forage to concentrate ratio condition, different dietary energy levels had little impact on the relative abundance of the major phyla and most of the major genera (*P* > 0.05), except for the genera SHD-231, *Succiniclasticum*, *Desulfovibrio*, and *Psychrobacter* (*P* < 0.05)*.* The relative abundance of the genus SHD-231 significantly decreased with the increase in dietary energy levels, and this genus was significantly more abundant (*P* < 0.05) in the L treatment than in the M and H treatments. The relative abundances of the genera *Succiniclasticum* and *Psychrobacter* significantly increased with the increase in dietary energy levels. The relative abundance of the genus *Succiniclasticum* was significantly higher (*P* < 0.05) in the H treatment than in the L treatment, and the relative abundance of the genus *Psychrobacter* was significantly higher (*P* < 0.05) in the H treatment than in the L and M treatments. The genus *Desulfovibrio* was significantly more abundant (*P* < 0.05) in the M treatment than in the H treatment.

### Relationship between the rumen bacteria and fermentation parameters

Ruminal fermentation parameters included the concentration of NH_3_-N and total VFA, and the molar proportions of acetate, propionate, butyrate, isovalerate, and valerate. These parameters above were not influenced by the dietary treatments (Table 4). The concentration of NH_3_-N and total VFA were significantly influenced by the age of the heifers, and interactions between dietary treatments and age were observed for the concentration of NH_3_-N and total VFA.

**Table 4:** Effects of dietary energy levels on the ammonia nitrogen and volatile fatty acids (VFA) of rumen samples in heifers aged 7 to 10 months

Items	Treatments			SEM	*P*		
	L	M	H		Treatment	Age	Treatment × Age
NH_3_-N (mg/dl)	15.15	15.17	15.60	1.339	0.9650	0.0204	0.0440
Total VFA (mM)	102.80	105.62	110.02	12.010	0.9131	0.0035	0.0304
Acetate (%)	67.61	67.37	67.89	0.0066	0.8586	0.1948	0.5483
Propionate (%)	20.12	19.80	20.03	0.0105	0.9766	0.4745	0.7426
Butyrate (%)	8.92	9.48	9.06	0.0033	0.4900	0.0630	0.9302
Valerate (%)	2.39	2.44	2.30	0.0012	0.7164	0.0607	0.2125
Isovalerate (%)	0.79	0.90	0.71	0.0009	0.4099	0.0588	0.5280

**Fig. 2:**
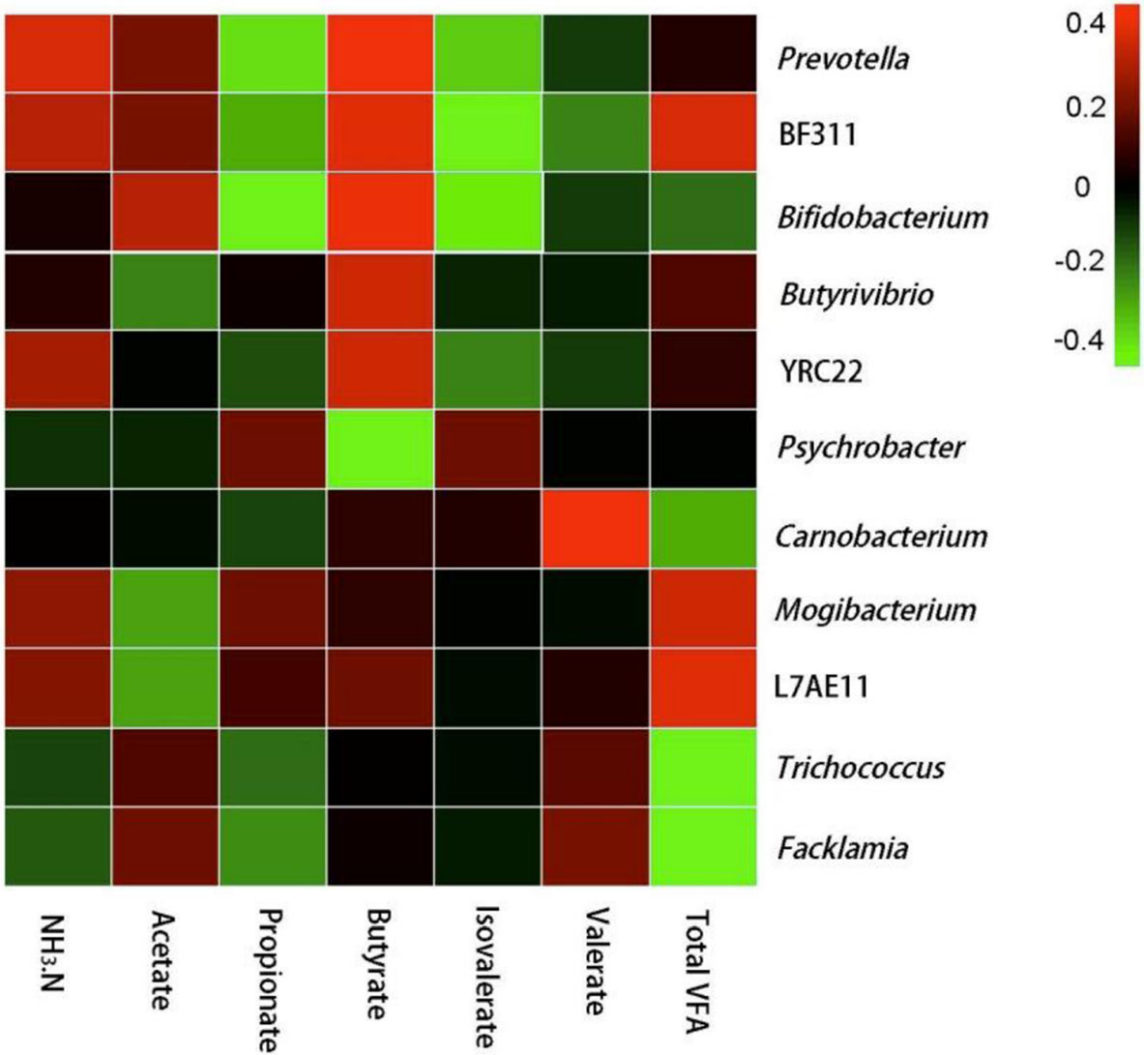
Correlation between the relative abundances of the rumen bacteria and the fermentation parameters(*P* < 0.05 was considered to be a significant correlation)

Correlation analysis was performed to identify the correlation between the relative abundances of the rumen bacteria and the fermentation parameters. The abundance of the rumen bacteria at the genus level and the concentrations of NH_3_-N and total VFA were regarded as significantly correlated with each other if *P* < 0.05. The NH_3_-N concentration was positively correlated with the relative abundances of the genera *Prevotella* and BF311 (Figure 2). The propionate molar proportion was negatively correlated with the relative abundances of the genera *Prevotella* and *Bifidobacterium*, and the isovalerate molar proportion was negatively correlated with the relative abundances of the genera BF311 and *Bifidobacterium*. The butyrate molar proportion was positively correlated with the relative abundances of *Prevotella*, BF311, *Butyrivibrio*, and *Bifidobacterium*, and was negatively correlated with *Psychrobacter* abundance. The valerate molar proportion was positively correlated with the relative abundance of *Carnobacterium*. The total VFA concentration was positively correlated with BF311 abundances, and was negatively correlated with *Trichococcus* and *Facklamia* abundances.

## Discussion

Rumen bacterial communities play a key role in the production performance and health of the host [33]. The objective of this study was to investigate the effects of different dietary energy levels, with the same forage to concentrate ratio, on the rumen bacterial community composition in Holstein heifers using barcoded pyrosequencing of hypervariable V3–V4 regions of the 16S rRNA gene, and to explore the relationship between rumen bacteria and ruminal fermentation parameters.

In this study, neither the alpha diversity nor the relative abundances of the main phyla and genera changed significantly among the different dietary treatments, indicating that, under the same forage to concentrate ratio of 50:50, dietary energy levels that varied by 8% did not affect the rumen bacterial community composition. Previous studies showed that, during transition from high-forage diets to high-grain diets, significant changes in rumen microbial population structure and diversity were detected, and a decrease in the amount of major cellulolytic bacterial species and an increase in the amount of amylolytic bacteria were observed [7, 9, 10]. Although different processing methods of ingredients affected the rumen microbial population structure [34, 35], under the same diet ingredients and processing conditions, the dietary forage to concentrate ratio was the main factor affecting the rumen microbial population structure. The *Firmicutes*, *Bacteroidetes*, and *Proteobacteria* were the dominant phyla in the rumen of heifers regardless of diet composition, and their relative abundances did not show significant changes between the groups. Similarly, at the genus level, the majority of the genera present in all groups with the relative abundance ≥0.1% were not affected by the different diets. The ruminal bacterial community composition in the current study was consistent with the known bacterial communities in dairy cattle, as bacteria from the phyla *Firmicutes*, *Bacteroidetes*, and *Proteobacteria* dominated the core bacterial community, regardless of feeding groups [20, 36].

Dietary changes have important impacts on rumen bacterial communities [10, 37, 38]. Fernando et al. [11] demonstrated that, when the dietary forage to concentrate ratio gradually increased from 80:20 to 60:40 or 20:80, the rumen bacterial population structure changed clearly, with the *Proteobacteria* increasing, and the *Firmicutes* and *Bacteroidetes* decreasing significantly. Petri et al. [7] investigated the rumen bacterial community in heifers during the transition from forage to high-grain diets and showed that the *Proteobacteria* increased and *Firmicutes* decreased as an excess of grain was introduced into the rumen. The increased abundance of *Proteobacteria* during high-grain diets is suggestive of an increased need for bacterial species that can metabolize the newly available fermentable carbohydrates [7, 11]. In this study, under the same forage to concentrate ratio of 50:50, the relative abundance of *Proteobacteria* increased, and *Firmicutes* and *Bacteroidetes* decreased with increasing dietary energy levels, but no significant changes were detected. Our observation of no significant changes in the abundance of most phyla may be due to the same forage to concentrate ratio in the diets, suggesting that the rumen bacterial community is mainly affected by forage to concentrate ratio, rather than the dietary energy level.

*Firmicutes* is the most abundant phylum accounting for more than 51% of the total sequences among different dietary treatments and is predominantly comprised of *Ruminococcus*, *Butyrivibrio*, *Succiniclasticum*, and *Carnobacterium*. *Firmicutes* are mainly composed of Gram-positive, low-G + C-content bacteria [39]. *Ruminococcus* is a fibrolytic organism that digests fiber and is predominantly present in high-fiber diets [1]. The abundance of *Ruminococcus* has been shown to gradually decrease during adaptation to a high-grain diet [10, 11]. In this study, the abundance of *Ruminococcus* was not affected by the three types of diet. The finding was not surprising, as the three diets comprised the same roughage composition and proportion. *Butyrivibrio* is known to be a fibrolytic bacterium, but it also has the ability to utilize starch and produce butyrate [11, 33], which is indicative that *Butyrivibrio* is able to utilize both fiber and starch to produce butyrate. This is consistent with the results of the positive correlation between the relative abundance of *Butyrivibrio* and the butyrate concentration. Previous studies found that the population of *Butyrivibrio* decreased in cattle on high-concentrate diets [11, 40]. In this study, the relative abundance of the genus *Butyrivibrio* was not influenced by the increasing dietary energy levels, which is suggestive that, under the same forage to concentrate ratio, the proportion of grain increasing from 15.42 to 27.85% had little influence on the abundance of *Butyrivibrio*. *Succiniclasticum* is a propionate-producing species and is known to produce propionate through succinate decarboxylation [41, 42]. In our study, the relative abundance of *Succiniclasticum* significantly increased in the high-energy diet group, consistent with findings that the abundance of *Succiniclasticum* increases in dairy cows fed high levels of concentrate [7, 43]. The genera *Carnobacterium*, which are regarded as lactic acid bacteria in the rumen [44], were not affected by the different diets. This may also be because the increasing range of grain in the diets is too small to cause changes. *Trichococcus* can metabolize a variety of complex organic compounds, such as multiple sugars, amino acids, polyols, and fatty acids, to produce lactate [45, 46], and *Trichococcus* also has the ability to degrade cellulose [47]. The negative correlation between the relative abundance of *Trichococcus* and the total VFA concentration in this study may be because of the decrease in ruminal pH limiting the growth of organisms that produce VFA, due to the increased amount of lactic acid produced by lactic acid bacteria. Unfortunately, the lactic acid concentration was not detected in the current study.

Sequence analysis of the reads from animals fed the three diets displayed a larger number of bacteria belonging to the phylum *Bacteroidetes* and this phylum was mainly composed of bacteria belonging to the genus *Prevotella*. The ruminal *Prevotella* species are a genetically and metabolically diverse bacterial group in rumen microbial communities, and they are numerically predominant in both the rumens of animals fed high-grain diets and those fed high-forage diet [48, 49]. *Prevotella* species are capable of utilizing starches, other non-cellulosic polysaccharides, and simple sugars as energy sources to produce succinate as the major fermentation end product [49]. In addition, ruminal *Prevotella* species, including members with hemicellulolytic and proteolytic activity [48], are considered to be involved in hemicellulose and pectin digestion [50], and protein or peptide metabolism [51, 52] in the rumen. In this study, the relative abundance of *Prevotella* was positively correlated with NH_3_-N and butyrate concentration. These results were consistent with previous observations reported by Chiquette et al. [53], who found that supplementation with members of the *Prevotella* as direct-fed microbials to dairy cows significantly increased the NH_3_-N, acetate, and butyrate concentrations in the rumen. The increased concentration of NH_3_-N is suggestive of an increased rate of proteolysis and amino acid metabolism in the animals. The increased concentration of butyrate is indicative of an increased rate of fiber fermentation.

*Proteobacteria* was the third most dominant phylum in the rumen, which was consistent with previous observations [20, 36]. The phylum is mainly composed of Gram-negative bacteria, which have highly diverse metabolic functions [54]. In this study, the *Proteobacteria* was composed of bacteria belonging to the genera *Psychrobacter* and *Acinetobacter*. The genus *Psychrobacter* has been regarded as containing psychrophilic organisms [55, 56]. However, members of this genus are highly varied in their cold adaptability and genomes, and have been isolated from various environments, such as Antarctic soil and seawater, Siberian permafrost, and the gut of marine fish [57–59]. Very little is known about the metabolism of this genus. Sun et al. [60] demonstrated that supplementing the diet with *Psychrobacter* enhanced intestinal digestive enzyme activities and improved feed utilization. In the present study, the relative abundance of the genus *Psychrobacter* was significantly higher in the high energy level diet than in the other two diets, and was negatively correlated with the butyrate concentration.

*Bifidobacterium* belonging to the phylum *Actinobacteria* represent Gram-positive, non-motile, and non-spore-forming bacteria [61]. Members of *Bifidobacterium* have extensive capabilities to metabolize dietary- as well as host-derived glycans, in particular starch and starch-like poly- and oligo-saccharides, such as pullulan, amylopectin, maltotriose, and maltodextrin [62, 63]. *Bifidobacterium* have been identified as the major lactic acid producing bacteria [64]. Lactic and acetic acids are the main metabolic end products produced by this genus [65]. The correlation analysis between the relative abundance of *Bifidobacterium* and the fermentation parameters showed that the former was positively correlated with butyrate concentration, and was negatively correlated with propionate and isovalerate concentration. The reported positive correlation between *Bifidobacterium* and butyrate concentration found by us in the present work is supported by previous study, in which *Bifidobacterium* was pointed to the involvement of butyrate production [66]. They believed that some *Bifidobacterium* strains were involved in butyrate production as a result of the secondary fermentation of the lactate and acetate by butyrate-producing bacteria through cross-feeding interactions [67–69]. Knowledge of substrate utilization and end products of members of the genera BF311 and *Facklamia* is limited, but correlation analysis showed that the metabolic capability of BF311 might be similar to the genus *Prevotella*, and the genus BF311 might participate in VFA metabolism. Future research of rumen bacterial community composition and functions is necessary to explain the relationship between the ruminal bacteria and the host.

## Conclusions

Under the same forage to concentrate ratio condition of 50:50, an 8% difference in dietary energy levels had no significant impact on rumen bacterial community composition in heifers. *Firmicutes*, *Bacteroidetes*, and *Proteobacteria* were detected as the dominant phyla in all treatments groups. Furthermore, the correlations between some genera of ruminal bacteria and the concentrations of NH_3_-N and VFA might be indicative that the ruminal fermentation parameters are strongly influenced by the rumen bacterial community composition. This study provides further information regarding the effects of different dietary energy levels on rumen bacterial community composition in heifers, and the relationship between rumen bacteria and ruminal fermentation parameters. This study may be of great interest for researchers investigating rumen microbial ecology, rumen fermentation function, and feed management.

## Abbreviations

bp: Base pair
ddH_2_O: Double distilled H_2_O
DNA: Deoxyribonucleic acid
dNTPs: Deoxyribonucleotide triphosphate
NH_3_-N: ammonia nitrogen
OTU: Operational taxonomic units
PCR: Polymerase chain reaction
PyNAST: Python Nearest Alignment Space Termination
RDP: Ribosomal Database Project
rRNA: Ribosomal ribonucleic acid
VFA: Volatile fatty acids
